# Open cervical lymph node biopsy for head and neck cancers: any benefit?

**DOI:** 10.1186/1758-3284-1-9

**Published:** 2009-04-29

**Authors:** Adeyi A Adoga, Olugbenga A Silas, Tonga L Nimkur

**Affiliations:** 1 Otorhinolaryngology Unit, Department of surgery, Jos University Teaching Hospital, PMB 2076, Jos, Plateau state, Nigeria; 2 Department of Pathology, Jos University Teaching Hospital, PMB 2076, Jos, Plateau state, Nigeria

## Abstract

**Background:**

Most patients with head and neck cancers in our environment present late and usually first to the general surgeons whose practice is to subject these patients to open cervical lymph node biopsy without a prior examination under anesthesia and endoscopic biopsy from the primary tumor site in order to obtain a histological diagnosis.

This paper presents the influence of open cervical lymph node biopsy on the clinical outcome of patients with head and neck cancers in our environment.

**Methods:**

This is a ten-year retrospective review of patients with head and neck cancers in the Jos University Teaching Hospital, Jos, Nigeria.

**Results:**

Eighty nine patients aged between 23 and 78 years had head and neck cancers with 38/89 (42.7%) patients having cervical lymphadenopathy at presentation and these initially presented to the general surgeons. Twenty six (68.4%) patients had open cervical lymph node biopsy and 12/38 (31.6%) patients had FNAB. Eleven (28.9%) patients presented to the otolaryngology unit 6 months after they presented to the general surgeons and 27 (71.1%) patients beyond 6 months. Nine deaths were recorded. Ten patients were lost to follow-up.

**Conclusion:**

All patients with head and neck lymphadenopathy who present to any physician for diagnostic examination should undergo formal ENT staging and FNAB to avoid the problems of tumor spread and the reduction in consequent prognosis.

## Background

Cervical lymph node metastasis can be the first manifestation of a carcinoma. The corresponding primary tumor is diagnosed in the ear, nose and throat region in 80% of cases and the bronchi or esophagus in 10% of cases [1]. Therefore, a thorough otorhinolaryngological examination supplemented by imaging studies and panendoscopy with directed biopsies of the suspicious primary sites must be undertaken for diagnosis, staging and grading [2].

In the assessment of patients with cervical lymph node enlargement, history and full clinical examination is essential [3]. This is supplemented by radiological diagnostic facilities like computerized tomographic (CT) scan, magnetic resonance imaging (MRI) and an examination under anesthesia [4,5]. Further assessment with fine needle aspiration biopsy (FNAB) and an open biopsy can help to confirm or refute the diagnosis of a head and neck malignancy [6,7].

Open cervical lymph node biopsy can alter patterns of lymphatic drainage for up to 1 year following surgery but does not portend a poor prognosis provided adequate and early treatment is subsequently given [8]. This however cannot be the same in our environment where it is common for patients to present late to the hospital. This late presentation is usually attributed to ignorance and poverty [9] and the final outcome is further worsened by the unavailability of proper diagnostic and therapeutic facilities to effectively manage these patients.

This paper focuses on the influence of open cervical lymph node biopsy on the outcome of patients with head and neck cancers in an ill-equipped tropical hospital setting.

## Methods

This is a ten-year (July 1998 to June 2008) retrospective study of patients presenting with various types of head and neck cancers to the otorhinolaryngology unit of the Department of Surgery, Jos University Teaching Hospital, Jos, Nigeria.

After obtaining approval from the Ethical Clearance Committee of the Jos University Teaching Hospital, the medical records of these patients were retrieved and analyzed for age, sex, nodal stage of disease at presentation, diagnostic protocol and treatment outcome.

The results are presented in a tabular form.

## Results

A total of 89 patients with head and neck cancers were managed aged between 23 years and 78 years. There were 65 (73.0%) males and 24 (27.0%) females with a male to female ratio of 2.7:1. Table 1 shows the representation of the various types of head and neck cancers seen. Thirty eight (42.7%) patients had cervical lymph node enlargement at presentation. Twenty nine (76.3%) patients had nasopharyngeal cancer, 6 (15.8%) had oropharyngeal cancer and 3 (7.9%) had sino-nasal cancers (Table 2).

**Table 1:** Frequencies of Head and neck cancers seen

**Cancer type**	**Frequency**	**Percentage**
Nasopharyngeal	46	51.7
Oropharyngeal	6	6.7
Hypopharyngeal	2	2.2
Sino-nasal	24	27
Laryngeal	7	7.9
Parotid	4	4.5
**Total**	**89**	**100**

**Table 2:** Patients with cervical lymph node enlargement at presentation

**Cancer type**	**Frequency**	**Percentage**
Nasopharyngeal	29	76.3
Oropharyngeal	6	15.8
Sino-nasal	3	7.9
**Total**	**38**	**100**

Twenty one (55.3%) patients presented to the general surgeons with N1 nodal disease, 12 (31.6%) patients with N2a nodal disease and 5 (13.2%) patients with N2b nodal disease. The duration between disease onset and presentation ranged from 8 to 15 months.

Four (4.5%) patients were able to afford CT scan.

Twenty six (68.4%) of the 38 patients who presented with cervical lymphadenopathy were subjected to open cervical lymph node biopsy by the general surgeons who were the first contact health personnel in our tertiary health center. The results from the pathologists which took a minimum of two weeks were reported as inconclusive and an advice for further evaluation of the patients to search for primaries in the head and neck. Twelve (31.6%) patients had FNAB with 9 positive for malignancy. All patients sustained scar following open biopsy, 15 (57.7%) had tumor spread.

Thirty eight patients presented to the otolaryngology unit and 37 of them had endoscopic biopsy of their primary tumors under general anesthesia (Table 3) and referred for radiotherapy. One patient with nasopharyngeal cancer died before he could have endoscopic biopsy. Eleven (28.9%) patients presented to the otorhinolaryngology unit after 6 months of first presentation to the general surgeons and 27 (71.1%) beyond 6 months. Nine deaths were recorded i.e. mortality rate of 10.1%. Five (55.6%) deaths were due to cancer, 3 (33.3%) from co-morbid conditions and in 1(11.1%), the cause of death was unknown. Ten patients were lost to follow-up, a common practice in our environment attributed to the inability of our patients to keep up with follow-up visits due to lack of finance.

**Table 3:** Histological diagnosis and staging in patients with cervical lymphadenopathy

	**Diagnosis**	**Clinical Stage**	**Path. Stage**	**Site**
1	Papillary SCC	3	4a	Oropharynx- R tonsil
2	Non-keratinizing SCC	3	4a	Nasopharynx- L wall
3	Well-differentiated SCC	3	3	Nasopharynx- L wall
4	Well-differentiated SCC	3	4a	Nasopharynx
5	Un-differentiated SCC	4a	4b	Nasopharynx- L wall
6	Un-differentiated SCC	3	4b	Oropharynx- soft palate
7	Rhabdomyosarcoma	3	3	Nasopharynx
8	Well-differentiated SCC	3	3	Nasopharynx- R wall
9	Non-Hodgkin's Lymphoma	3	3	L max. sinus
10	Non-Hodgkin's Lymphoma	4b	4b	Oropharynx- L tonsil
11	Un-differentiated SCC	4a	4b	Nasopharynx
12	Keratinizing SCC	3	3	Nasopharynx
13	Well-differentiated SCC	3	3	Nasopharynx- R wall
14	Poorly-differentiated SCC	3	4a	Nasopharynx
15	Well-differentiated SCC	3	3	Nasopharynx- R wall
16	Well-differentiated SCC	3	3	Nasopharynx- L wall
17	Adenocarcinoma	4a	4b	R nose
18	Well-differentiated SCC	3	4a	Nasopharynx
19	Non-Hodgkin's Lymphoma	3	3	Oropharynx- R tonsil
20	Lymphoepithelioma	3	3	Nasopharynx
21	Un-differentiated SCC	3	4a	Nasopharynx- L wall
22	Well-differentiated SCC	3	3	Nasopharynx
23	Non-keratinizing SCC	3	4a	Nasopharynx
24	Well-differentiated SCC	3	3	Nasopharynx
25	Poorly-differentiated SCC	3	3	Nasopharynx- R wall
26	Poorly-differentiated SCC	4a	4b	Nasopharynx- R wall
27	Well-differentiated SCC	3	3	Nasopharynx- L wall
28	Poorly-differentiated SCC	3	4b	Oropharynx- R tonsil
29	Non-keratinizing SCC	3	3	Nasopharynx
30	Non-Hodgkin's Lymphoma	4a	4a	Oropharynx- L tonsil
31	Well-differentiated SCC	3	3	Nasopharynx- L wall
32	Non-keratinizing SCC	3	4b	Nasopharynx
33	Un-differentiated SCC	3	4a	Nasopharynx- R wall
34	Well-differentiated SCC	3	3	Nasopharynx
35	Unknown	3		
36	Poorly-differentiated SCC	4b	4b	R ethm.sinus
37	Un-differentiated SCC	4a	4b	Nasopharynx
38	Well-differentiated SCC	3	3	Nasopharynx

Key: R-right, L-left, SCC-squamous cell carcinoma, Ethm.-ethmoid, Path.-pathological

## Discussion

An important prognostic factor for cancers of the head and neck is the presence or absence, level and size of metastatic neck disease [10].

Both tumor and patient factors affect the pattern of spread of malignant disease to the neck [11]. The primary site of a tumor is important, with some sites having a high incidence of metastases than others at presentation.

Lindberg in 1972 was able to establish the possibility of predicting the site of a primary tumor in the head and neck based on the distribution of cervical metastasis [12]. Following this, the Memorial Sloan-Kettering Hospital in 1981 published 7 levels or regions in the neck which contain groups of lymph nodes that represent the first echelon sites for metastasis from head and neck primary tumors [13]. For example, the nasopharynx, nasal cavities and paranasal sinuses drain via the junctional nodes into the upper deep cervical lymph nodes in levels II-III... e.t.c.

The management of a patient with cervical lymph node enlargement starts with a history of the disease, full clinical examination, and radiological investigations such as CT scan, MRI, ultrasound and radionuclide scanning [3,4]. These are supplemented by an examination under anesthesia and panendoscopy to look for the primary site of tumor with biopsies of suspicious tumor sites [5].

Fine needle aspiration biopsy is preferable to open biopsy of a cervical lymph node for the reasons that there is no tumor spread, no inconvenient scar to distort future surgical intervention, no delay between diagnosis and treatment and its simplicity. When a diagnosis of malignancy cannot be made by needle biopsy, then an open biopsy can be done provided it can be followed by a frozen section and a concomitant definitive neck dissection if peroperative positive histological diagnosis is obtained [14]. Open cervical lymph node biopsy can alter patterns of lymphatic drainage for up to 1 year following surgery [8] and creates a scar which distorts future surgical intervention therefore altering the outcome of treatment [14].

In our environment, the interplay of several factors contributes to the poor outcome in the management of head and neck cancer patients. These factors include late patient presentation, inaccessible health facilities and delay in the availability of histopathology results following biopsies.

Our study shows that our head and neck cancer patients presented late to the hospital which is a common feature here attributed to poverty and ignorance [9].

All the patients in our study were referred to the general surgeons by health workers from neighboring primary health centers which explains why they were the first contact health personnel in our tertiary health center.

Facilities for frozen section are not available in our center and delay in getting histopathology results from the open cervical biopsies further compounds our patients' problems as their tumors and disease process progresses further with advanced nodal diseases on presentation to the otorhinolaryngologist who is left with little or no help to offer these patients at the time they present.

Even though FNAB done for 12 of our patients was able to detect malignancy in 9 patients, all had examination under anesthesia to detect the site of primary tumor and to be able to get biopsy material for histological diagnosis. Knowing the site of primary tumor is essential in planning treatment. However, in this study it is not clearly stated by the general surgeons the criteria used for subjecting some patients to open biopsy and others to FNAB.

Majority of our people cannot afford the cost of diagnostic facilities like CT scan. This is further buttressed by the fact that only four patients in our series could afford it- patients number 24, 25, 31 and 38 as seen on Table 3 below. These patients benefitted from this radiological diagnosis and are still alive following treatment.

Nine deaths were recorded in our series. These are shown on Table 3 to be patients' number 5, 6, 10, 11, 26, 32, 35, 36 and 37 who had advanced disease. Three of these also had co-morbid medical conditions (hypertension and diabetes mellitus) and 1 (patient number 35) died before he could have endoscopic biopsy.

The mortalities recorded in our series were all as a result of interplay of the above mentioned factors. To overcome these, we recommend that all head and neck cancer patients especially those with cervical lymph node enlargement on presentation should have an examination under anesthesia and endoscopic biopsies taken from all suspicious primary tumor sites to obtain histological diagnosis rather than be subjected to open cervical lymph node biopsy. This enables planning for early and proper treatment. This can be done by the first contact health personnel or a referral to the otorhinolaryngologist or head and neck oncologist to avoid delay in patient management and to institute early treatment.

## Conclusion

While we await improvements in the diagnostic and therapeutic facilities for the care of patients with head and neck cancers in our environment, we recommend that all patients with head and neck lymphadenopathy who present to any physician for diagnostic examination should undergo formal ENT staging and FNAB to avoid the problems of tumor spread and the reduction in consequent prognosis.

## Competing interests

The authors declare that they have no competing interests.

## Authors' contributions

AAA was the principal surgeon, performed literature search and prepared the manuscript. OAS prepared and read the slides and also reviewed the manuscript. TLN assisted in the surgeries, post-operative management of patients and manuscript review.
